# Chiari Formation or Malformation? Trends in the Pathophysiology and Surgical Treatment of an Ever-Elusive Entity

**DOI:** 10.3390/brainsci14101006

**Published:** 2024-10-05

**Authors:** Massimiliano Visocchi, Alberto Benato, Davide Palombi, Francesco Signorelli

**Affiliations:** 1 Operative Unit and Academic Research Center on Surgical Approaches to the Craniovertebral Junction, Università Cattolica del Sacro Cuore, 00168 Rome, Italy; massimiliano.visocchi@policlinicogemelli.it; 2 Fondazione Policlinico Universitario “A. Gemelli” IRCCS, 00168 Rome, Italy; davidepalombi@hotmail.it (D.P.); francesco.signorelli@policlinicogemelli.it (F.S.); 3 Faculty of Medicine, Università Cattolica del Sacro Cuore, 00168 Rome, Italy

**Keywords:** Chiari malformation, craniovertebral junction, syringomyelia, review, innovation, minimally invasive spine surgery

## Abstract

**Background**: Chiari malformation type 1 (CM1) remains a complex neurosurgical condition with ongoing debate regarding its optimal management. **Methods**: This narrative review examines key controversies surrounding the pathophysiology, surgical indications, and treatment strategies for CM1. **Results**: We highlight the challenges posed by the wide spectrum of CM variants and the evolving understanding of its association with syringomyelia, basilar invagination, and craniovertebral instability. Emerging surgical techniques, including minimally invasive approaches and the use of new technologies such as endoscopes and exoscopes, are evaluated for their potential to improve outcomes. Recent consensus guidelines are also discussed. **Conclusions**: The need for individualized treatment plans for CM1 is emphasized, with special focus put on the connection between novel pathophysiological insights, technological advancements and opportunities for a more nuanced surgical management. Further research is necessary to establish solid foundations for more individualized treatments.

## 1. Introduction

The optimal surgical management of Chiari malformation represents one of the ongoing neurosurgery debates [1]. The evasiveness of the condition, representing a spectrum of anatomical, clinical, and radiological features, has been an obstacle to providing univocal, precise treatment and follow-up guidelines [2]. In this paper, we aim to explore the current controversies in CM1 management, including pathophysiology, surgical indications, techniques, and the emerging role of new technologies [3]. By synthesizing recent research findings and expert opinions, we seek to provide a comprehensive overview of the current state of CM1 management and highlight areas where further investigation is needed to develop well-founded therapeutic guidelines.

## 2. Methods

With this work, we provide a narrative overview of the literature on CM1 with a special focus on the ongoing controversies regarding its pathophysiology and innovations in its management. We screened the MEDLINE database using the following search string: (Chiari OR CM1 OR CIM OR “tonsillar herniation” OR syringomyelia) AND (pathophysiology OR management OR “minimally invasive” OR endoscopic OR technolog* OR innovation). We excluded from the search all articles written in languages other than English and studies conducted on non-human animals. Three authors (A.B., D.P., and F.S.) screened the 5874 results. Under the guidance of the senior author (M.V.), a selection of relevant papers providing an overview of the aforementioned controversies and innovations was made.

## 3. The History of Chiari Malformation(s)

In 1891, Hans Chiari described a group of congenital hindbrain anomalies that would later bear his name [4]. Chiari initially classified these malformations into three types: Chiari I, II, and III. Four years later, he added Chiari IV to the classification [5]. Since then, researchers have proposed new classifications for variants that do not fit Chiari’s original descriptions, such as Chiari 0, Chiari 1.5, and Chiari 3.5 malformations (Table 1) [6]. Each type has distinct anatomical characteristics, some extremely rare and incompatible with life, like Chiari 3.5. Due to the rarity and uniqueness of these conditions, some physicians may be unfamiliar with the newer classifications. Moreover, some debate still exists regarding the genuine individuality of some of the described forms of CM, such as Chiari 1.5 [7]. Chiari 1.5 has been described as a descent of both cerebellar tonsils and brainstem sagging, associated with CVJ anomalies such as basilar invagination, atlantooccipital assimilation, etc. [8].

However, according to some authors, Chiari 1.5 is just part of the spectrum of Chiari 1 [7]. Notably, the definition of Chiari IV malformation has been further clarified, and it is crucial to use this term consistently with Chiari’s original description, which involves an occipital encephalocele containing supratentorial contents. Due to the sporadic nature of anomalies such as CM3, CM3.5, CM4, and CM5, and the absence of precise embryological investigations, these malformations may be unrelated to the more common CM0, CM1, CM1.5, and CM2 [9].

**Table 1:** Summary of the described variants of Chiari malformation.

Type	Abbreviation	Author/Year	Main Characteristic
CHIARI 0	CM0	Iskandar et al., 1998 [10]	Posterior fossa crowding without tonsillar herniation (TH)
CHIARI 0.5	CM0.5	Morgenstern et al., 2020 [11]	Ventrolateral tonsillar wrapping
CHIARI 1	CM1	Chiari, 1891 [4]	Tonsillar herniation (>3–5 mm)
CHIARI 1.5	CM1.5	Tubbs et al., 2004 [12]	Cerebellar and brainstem herniation ± basilar invagination, platybasia, Klippel–Feil syndrome, …
CHIARI 2	CM2	Chiari, 1891 [4]	Spina bifida, cerebellar–brainstem herniation
CHIARI 3	CM3	Chiari, 1891 [4]	Cerebellar–cervical encephalocele
CHIARI 3.5	CM3.5	Fisahn et al., 2016 [13]	Cerebellar–cervical encephalocele connected to foregut
CHIARI 4	CM4	Chiari, 1896 [5]	Cerebellar hypoplasia or aplasia without TH
CHIARI 5	CM5	Tubbs et al., 2012 [14]	Cerebellar aplasia and occipital lobe herniation

## 4. Theories and Controversies on Chiari Malformation Pathogenesis

A search on PubMed reveals 3143 results on the pathogenesis of CM1. Various theories have been proposed (Table 2):

It is important to distinguish a fundamentally different mechanism of tonsillar herniation, i.e., spontaneous intracranial hypotension. In this case, tonsillar descent is generated by an opposite gradient that “sucks down” intracranial contents into the foramen magnum [19]. The treatment of this condition is fundamentally different and based on repairing the leak (overt or concealed) in the spinal thecal sac that generates the pressure gradient.

Additionally, the association between Chiari malformations (CMs) and syringomyelia is frequent, although percentages reported in the literature vary widely [20]. The pathophysiology underlying this association has been studied for a long time, and various theories have been developed [21]. The classic theory states that an alteration in CSF flow at the level of the foramen magnum, produced by tonsillar herniation and encroachment of the foramen magnum itself, generates a pressure gradient between the cisternal and intramedullary compartments, favoring the penetration and accumulation of CSF in the spinal cord [22].

### 4.1. Formation or Malformation?

One of the most debated topics is whether CM1 always represents a pathological entity, i.e., if it could be a spectrum ranging from quasi-physiological entities to markedly pathological conditions [23]. According to this latter perspective, in some individuals, the anatomical configuration of the cerebellar tonsils and the foramen magnum might fall within a spectrum of normal variation rather than constituting a definitive pathology. In the literature, multiple studies have associated CM1 with a reduction in posterior fossa volume and other anomalies of the occipital bone (theory 3 in the paragraph above) resulting from an abnormal development of the occipital somite [24]. However, some studies have challenged the idea that a reduced posterior fossa volume is inherently pathological, showing an intrinsic variability in various measurements in the average and CM1 populations. For instance, Roller et al. found demographic confounders in volumetric MRI analyses, suggesting that a smaller posterior fossa does not necessarily underlie pathology in adults with CM1 [25]. Similarly, Sgouros et al. described variability in posterior fossa volume among children with CM1, stressing that not all anatomical variations correlate with clinical symptoms [26]. Furthermore, Tubbs et al. conducted a volumetric analysis in a family spanning four generations with CM1, indicating that such anatomical features might be familial traits in the normal range rather than malformations [23]. This view challenges the traditional paradigm, suggesting that asymptomatic CM1 could be considered an anatomical variant, potentially resulting from evolutionary adaptations, rather than a pathological malformation requiring correction [27]. This hypothesis underscores the importance of a cautious, individualized approach to diagnosing and treating CM1, emphasizing the need to distinguish between “simple” forms that could be interpreted as anatomical variations and overt pathological scenarios that necessitate intervention [28,29].

### 4.2. Atlantoaxial Instability and Chiari Malformation

CM1 has a well-known association with malformations of the cranial base and craniovertebral junction, characterized by variable degrees of instability [30]. In recent years, the association between CM1 and instability of the craniovertebral junction has been at the center of a debate originating from a statement by Goel (2015) [18]. Goel’s theory suggests that CM with or without syringomyelia is essentially caused by instability at the craniovertebral junction, which links conditions such as basilar invagination, Chiari malformation, and syringomyelia [18]. In this theoretical framework, tonsillar herniation is not seen as the primary pathological element of CM1 but rather as a compensatory mechanism, acting as an airbag that protects the bulbomedullary junction from dynamic compression [30]. The alteration in CSF dynamics leading to syringomyelia would also be connected to this phenomenon. Goel’s theory implies that a degree of CVJ instability, overt or concealed, is always present in CM1 patients.

Various authors and groups, such as Jian et al. (2010) and Menezes (2008), have clearly illustrated the role of CVJ fixation in treating CM1 associated with CVJ instability or malformations, resulting in better patient outcomes [30,31,32]. Subsequent studies by Salunke et al. (2012) and Wang et al. (2011) have shown as well that, with accurate indications, stabilization of the atlantoaxial joint leads to significant symptomatic improvement and reduced recurrence rates [33,34].

However, Goel advocates for atlantoaxial fixation in all cases of CM with or without syringomyelia, irrespective of the presence or absence of basilar invagination or other alterations in CVJ structure/mechanics, suggesting that some form of CVJ instability is always present in CM1, as stated above [18]. The rationale is that stabilizing this joint would lead to a regression of tonsillar herniation and would solve the alteration in CSF flow, alleviating symptoms and preventing the progression of the condition.

**Figure 1:**
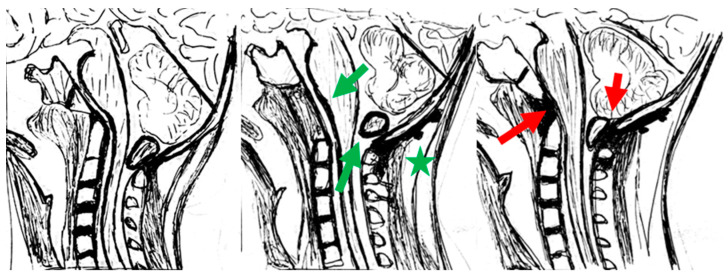
Schematic drawings reproducing the imaging from the personal case also described in Visocchi et al., 2011 (© European Spine Journal, Springer, 2011) [35]. (**Left**): preoperative picture, with basilar invagination with ventral brainstem compression and tonsillar herniation. (**Center**): immediate postoperative picture, showing successful ventral decompression and regression of tonsillar herniation (green arrows) with occipitocervical fusion (green asterisk); (**Right**): picture at the 1-year follow-up, showing partial regrowth of the odontoid and lower clivus, recurrence of ventral compression, and recurrence of tonsillar herniation (red arrows) despite the presence of occipital–cervical fixation.

This approach sparks debate within the neurosurgical community. The main consensus is that not all cases of CM are due to atlantoaxial instability and that the need for fixation procedures should be carefully evaluated. Although some studies and case reports support Goel’s hypothesis, indicating improvements in symptoms and imaging findings post-fixation, some published cases refute his theory. For example, the senior author of this paper (M.V.) has previously published the case of an adolescent that had CM1 associated with basilar invagination. The patient was treated with transoral decompression and occipitocervical fixation, with a resolution of CM. However, a few months later, extensive bony regeneration occurred ventrally, recreating anterior compression, with the reappearance of tonsillar herniation (Figure 1). Thus, CM recurred in this patient despite a fixed and stable OC junction. This suggests an equally important role of ventral compression in the pathophysiology of CM [35].

Despite the ongoing debate, Goel’s theory has offered a new perspective for understanding the pathophysiology of CM and has encouraged a re-evaluation of traditional treatment paradigms. The theory also stresses the importance of considering craniovertebral junction stability in diagnosing and treating patients with CM. More extensive research and experience are needed to establish the efficacy and safety of atlantoaxial fixation as a routine treatment for CM.

## 5. Clinical Patterns of Chiari Malformations

A search on PubMed yields 1049 results solely on the clinical patterns of Chiari malformations. Besides the textbook presentation of occipital headaches, exacerbated by increased intracranial pressure, symptoms can include cranial nerve or brainstem dysfunction. These symptoms can affect both young and adult patients and are associated with more complex deformities like ventral compression due to basilar invagination, retroflection of the dens, and craniocervical instability [36]. Patients with syringomyelia can exhibit a wide range of symptoms ranging from spinal cord dysfunction to the development of dysfunctional postures and spine deformities [37].

Neuropsychological evaluations of patients with Chiari malformations have shown poorer executive functioning, verbal fluency, spatial cognition, language, verbal memory, processing speed, facial recognition, and theory of mind compared to control groups [36,38]. These findings suggest a generalized cognitive deficit associated with Chiari malformations, highlighting the need to address physical and cognitive consequences [36].

The complexity of the spectrum of these clinical manifestations and their intricated relationship with the radiological patterns of CM is part of the reason why the optimal treatment strategy for CM is still a matter of significant debate in the neurosurgical community.

## 6. Recent Consensus Publications, Surgical Indications, and Controversies

Recently, two consensus papers were published, addressing the various aspects of CM1 management in adults and children, mainly focusing on surgical indications and options [1,2]. These papers brought together experts from diverse backgrounds to synthesize the latest research findings and clinical experiences, aiming to establish more uniform guidelines for treating CM [39].

The first paper [2] clarified the surgical indications for adult patients with Chiari malformation (CM). Experts unanimously agreed (100%) on the necessity of surgical intervention for symptomatic patients, i.e., those with severe, therapy-resistant headaches and neurological signs. Here, surgery aims to relieve symptoms by decompressing the cerebellar tonsils, which is essential to prevent further deterioration.

Conversely, experts unanimously agreed (100%) that surgery should be avoided in asymptomatic patients with isolated CM1, as the risks outweigh the potential benefits in the absence of clinical symptoms. However, follow-up with neurological evaluation and whole-neuraxis MRI is mandatory for these patients, with scheduling based on clinical and MRI findings.

In CM1 with syringomyelia, surgery is indicated based on the features of syringomyelia (holocord syringomyelia, a Vaquero Index > 0.5, eccentric syringes, etc.), a worsening clinical or MRI picture, or syringomyelia–syringobulbia with spinal or bulbar signs. In asymptomatic CM1 with syringomyelia, surgical decisions are less linear. Neurophysiological studies, including SEPs, BAEPs, and MEPs, may detect subclinical dysfunctions and provide objective evidence that could indicate the need for surgery or justify continued monitoring. Polysomnography is recommended for CM1 patients with reported or suspected sleep apnea, regardless of syringomyelia’s presence.

Another consensus paper was oriented toward the management of CM in pediatric patients [1]. The authors concluded that surgery is not indicated in asymptomatic children with incidental Chiari malformation type 1 (CM1) without syringomyelia (94%). Surgery is recommended when syringomyelia is present in asymptomatic patients if the syrinx exceeds 5–8 mm or demonstrates enlargement during follow-up (even if it is smaller than this threshold). This statement reached a consensus of 82%. A significant agreement was also reached in stating that surgery does not improve seizure disorders associated with CM1. However, surgery is not indicated in such children if no apparent CM1 symptoms are present, as it will not improve the overall clinical picture.

**Figure 2:**
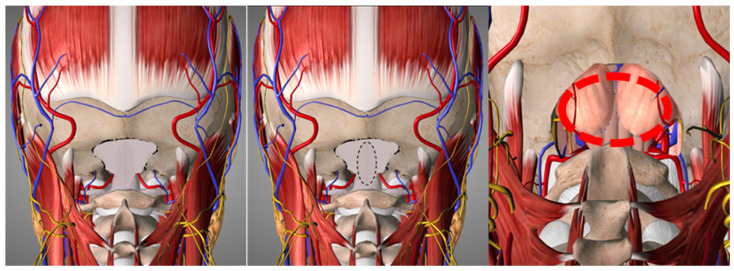
Three-dimensional drawings represent the main surgical steps in treating CM and associated syringomyelia. (**Left**): posterior fossa osteo-ligamentous decompression (PFD); (**Center**): osteo-ligamentous decompression with duraplasty (PFDD); (**Right**): coagulation/resection of the herniated tonsils (red circle). C1 laminectomy (not portrayed) can be added for additional decompression and enlarging the extent of the duraplasty. Extension of the decompression to the C2 posterior arch is not recommended, so as not to destabilize the CVJ. The Essential Anatomy software (3D4Medical, Elsevier) obtained the images.

As regards surgical techniques, recommendations were the following, with a consensus oscillating between 80 and 86% (in pediatric patients) and 75 and 87% (in adult patients):

- Osteo-ligamentous decompression alone (Figure 2) can be considered in pediatric patients without syringomyelia (80%) but is not recommended in adults (75%). This less invasive technique reduces the risks associated with the opening of the dura. Bony decompression should be comprehensive and always extended to C1; C2 posterior elements should not be resected to reduce the risk of iatrogenic CVJ instability.
- When syringomyelia is present, duraplasty (Figure 2) should always be performed. Autologous patches or allografts should be preferred over synthetic patches.
- Tonsillar coagulation and resection (Figure 2) can be considered when the tonsillar descent is significant and in recurrent/residual syrinx cases.

Both consensus meetings stressed the need for ongoing research and integration of new diagnostic tools and surgical techniques, which continue to evolve [40].

Together with these papers, it is worth mentioning that in 2021, the Congress of Neurological Surgeons (CNS) conducted a systematic review to synthetize recommendations on the optimal management of CM1. The authors suggest that either PFD or PFDD can be initial treatments for symptomatic CM1, with or without syrinx. In cases with syrinx, reducing or resecting cerebellar tonsil tissue may alleviate symptoms by improving CSF flow and reducing syrinx size [41].

### 6.1. Controversies in Syringomyelia Management

As stated above, PFDD is recommended as the primary treatment for CM1-associated syringomyelia [1,2]. According to some pathophysiological hypotheses, abnormalities of the arachnoid (membranes, bands, etc.) could contribute to the altered CSF dynamics that promote and sustain the development of syringomyelia [42]. However, a meta-analysis suggested that preserving the arachnoid in adults treated with PFDD results in better clinical outcomes and fewer complications than techniques involving arachnoid dissection [16,43]. Thus, arachnoid preservation is recommended to minimize complications and maintain effective decompression [44].

The rationale behind preserving the arachnoid may be that it mitigates the impact of CSF pressure waves on the dural suture, thus reducing CSF-related complications, which can occur in up to 21.8% of CM1 patients postoperatively [45,46,47]. Intradural exploration with tonsillar shrinkage and additional procedures, such as a fourth ventricle to spinal subarachnoid space stent (FVSSS), may have a role in treating a refractory syrinx. The management of recurrent syringomyelia is much less standardized, and various more invasive treatment modalities have been proposed in the literature to obtain a more effective syrinx decompression in these cases [48,49,50,51]. Some options include the following:

- Concomitant FMD with syringo-subarachnoid shunt: it aims at obtaining a more effective decompression of the syrinx by creating an artificial communication with the subarachnoid space, although it comes with an increased risk of complications [51,52].
- Syringopleural and syringoperitoneal shunts: these procedures can help manage persistent or progressive syrinx but are associated with risks such as shunt malpositioning and spinal cord tethering [53,54].

Further studies are needed to provide more precise and standardized indications for these procedures, as well as define which may be more indicated based on the individualized features of the patient.

### 6.2. Management of Associated Hydrocephalus

Since the original description of CM by Hans Chiari, an association between CM and hydrocephalus has been noted, manifesting in 7–10% of patients [55]. In the original description, tonsillar herniation derived from increased intracranial pressure related to the hydrocephalus [9]. However, in most cases, hydrocephalus is thought to be an extreme consequence of the global alteration in CSF dynamics that characterizes CM. Hydrocephalus in CM patients can exacerbate symptoms and complicate the clinical picture [56]. Surgical intervention with posterior fossa decompression theoretically restores normal CSF flow and thus should alleviate both CM and CM-related hydrocephalus [55,57]. However, the relationship between these conditions remains complex. Studies have shown that while successful decompression can lead to the improvement or resolution of hydrocephalus in many cases, in some patients, hydrocephalus may only stabilize rather than fully resolve [58]. Treatments that directly address hydrocephalus, such as ETV, have been shown in some studies to have a positive impact on tonsillar herniation as well and have been recommended as a first-line treatment [57].

### 6.3. Filum Terminale and Chiari I Controversy

The section of the filum terminale is traditionally used to treat tethered cord syndrome (TCS) and alleviate the abnormal tension on the spinal cord produced by this condition. In addition, occult tethered cord syndrome (OTCS) has been described as a condition where symptoms of TCS appear without radiological evidence of tethering. This has been controversially identified in CM1 patients [59].

PFD or PFDD represents the standard and accepted treatments for CM1. However, some authors have suggested that CM1 might be linked to OTCS and that SFT could be beneficial [60].

In 2011, Massimi et al. discussed elements in favor and against the existence of OTCS in CM1 patients. They concluded that the evidence to support this claim is inconsistent [61]. The authors, therefore, concluded that the “caudal traction theory”, which suggests that CM1 might result from spinal cord traction from below, is not supported by evidence and can be considered outdated [62].

The paper references studies that reported improvement in CM1 symptoms following SFT in some patients. However, the authors criticize these studies for methodological issues and lack of long-term data [63].

The authors conclude that no substantial evidence supports a connection between CM1 and OTCS. This was also confirmed by a systematic review by Milano et al., who stressed that there is no scientific support for FT sectioning in patients with CM without evidence of a tethered cord [64].

As part of this controversy, in 2012, the Italian Society of Neurosurgery (Sinch, Società Italiana di Neurochirurgia) released a statement against the sectioning of the filum terminale as a treatment for CMI in cases where there is no clear clinical and radiological evidence of a tethered spinal cord.

### 6.4. Surgical Innovations and Other Treatments

Minimally Invasive Spine Surgery (MISS) techniques aim at treating spine conditions with minimal disruption of normal tissues [65,66]. By reducing damage to muscles, bone, and connective tissues, such techniques can lead to quicker recovery, less postoperative pain, and shorter hospital stays than traditional open spine surgery [67,68,69]. Various studies have been published in the past 15 years illustrating the potential of MISS techniques in treating CM1 (Table 3) [67,70].

A similar trend is the introduction of endoscopic techniques for CM1 treatment, offering a potentially less invasive alternative to traditional open surgery [71,72]. Studies have highlighted the benefits of endoscopic techniques, including decreased postoperative pain, fewer complications (such as CSF leaks), and quicker recovery times [72,73].

Regarding improvements in intraoperative visualization, developing three-dimensional exoscopes could provide an alternative to traditional microscopes, offering comparable or superior image quality and fewer constraints. The exoscope enhances surgeon ergonomics and comfort and is particularly beneficial during lengthy procedures or when working in unfavorable surgical corridors, especially in the posterior cranial fossa [75]. Although the literature on using exoscopes in CM1 treatment has still to develop, this technology could show promise as a potential adjunct or replacement for conventional neurosurgical microscopes [74].

## 7. Conclusions

In conclusion, while there is consensus among experts on certain critical aspects of CM1 treatment, the field remains open to debate, with significant potential for future advancements and innovation. Acquisition of a deeper understanding of CM1 pathophysiology, along with the integration of novel surgical techniques, will be critical to driving these advancements. Through this review, we aimed to provide an overview of the current progress and the emerging possibilities in this evolving field.

## Figures and Tables

**Table 2:** Summary of the main theories on CM pathogenesis.

Mechanism	Description	Proposed By
Lack of perforation of the rhombencephalic roof	Incomplete perforation of the rhombencephalic roof during embryonic development leads to Chiari malformation.	Gardner [15]
Piston-like motion with or without intracranial pressure increase	The piston-like motion of the cerebellar tonsils, possibly exacerbated by increased intracranial pressure, causes the tonsils to descend into the foramen magnum.	Oldfield [16]
Underdevelopment of the occipital bone and posterior cranial fossa	Underdevelopment of the occipital bone and small posterior cranial fossa results in inadequate space for the cerebellum, leading to its herniation.	Nishikawa [17]
Atlantoaxial instability generates a protective/compensatory mechanism	Instability at the atlantoaxial joint triggers a protective or compensatory mechanism, leading to cerebellar tonsillar descent.	Goel [18]

**Table 3:** Summary of the leading publications regarding MISS treatments for CM.

Study	N. of Patients	Age	Skin Incision Size	Instrumentation Used	Complications (n. Reported)
Di et al., 2009 [71]	26	18 m–16 y	2 cm	Long-rod rigid endoscope	Bacterial Meningitis (1)
Deng et al., 2010 [72]	21	11 ± 5 y	2.5 cm	Long-rod rigid endoscope	Aseptic meningitis (1)
Gandhi et al., 2013 [65]	4	NR	2–2.5 cm	Tubular retractor	Aseptic meningitis (1)
Pazkaban et al., 2017 [73]	6	21–41 y	3–4 cm	Speculum retractors	None
Ratre et al., 2017 [66]	15	26–48 y	2.5–3 cm	Long-rod rigid endoscope	None
Khalessi et al., 2017 [74]	2	NR	NR	3-D exoscope	None
Kotil et al., 2018 [68]	61	15–58 y	1.5 cm	Speculum retractors	Superficial cutaneous infection (1)
Zagzoog et al., 2019 [69]	22	19–71 y	NR	METRx tubular retractor	VP shunt for hydrocephalus (1)
Tyagi et al., 2023 [70]	1	47	2–2.5 cm	METRx tubular retractor	None
Costa et al., 2024 [67]	10	25–62 y	3–4 cm	Mars3VL retractor	None

## Data Availability

No new data were created or analyzed in this study.

## References

[1] Massimi L., Peretta P., Erbetta A., Solari A., Farinotti M., Ciaramitaro P., Saletti V., Caldarelli M., Canheu A.C., Celada C. (2022). Diagnosis and Treatment of Chiari Malformation Type 1 in Children: The International Consensus Document. Neurol. Sci..

[2] Ciaramitaro P., Massimi L., Bertuccio A., Solari A., Farinotti M., Peretta P., Saletti V., Chiapparini L., Barbanera A., Garbossa D. (2022). Diagnosis and Treatment of Chiari Malformation and Syringomyelia in Adults: International Consensus Document. Neurol. Sci..

[3] Visocchi M., Mattogno P.P. (2020). Chiari Malformations: The Impact of Clinical and Surgical Features on a Journal Reviewer’s Evaluation Protocol. J. Neurosurg. Sci..

[4] Chiari H. (1891). Ueber Veränderungen des Kleinhirns infolge von Hydrocephalie des Grosshirns ^1^). Dtsch. Med. Wochenschr..

[5] Chiari H. (1896). Über Veränderungen des Kleinhirns, des Pons und der Medulla oblongata in Folge von Congenitaler Hydrocephalie des Grosshirns.

[6] Azahraa Haddad F., Qaisi I., Joudeh N., Dajani H., Jumah F., Elmashala A., Adeeb N., Chern J.J., Tubbs R.S. (2018). The Newer Classifications of the Chiari Malformations with Clarifications: An Anatomical Review. Clin. Anat..

[7] Liu W., Wu H., Aikebaier Y., Wulabieke M., Paerhati R., Yang X. (2017). No Significant Difference between Chiari Malformation Type 1.5 and Type I. Clin. Neurol. Neurosurg..

[8] Benato A., Offi M., Bianchi F., Visocchi M., Tamburrini G. (2023). Atlas Invagination through the Foramen Magnum: Expanding the Spectrum of Craniovertebral Junction Malformations. Eur. Spine J..

[9] Bianchi F., Montedoro B., Frassanito P., Massimi L., Tamburrini G. (2023). Chiari I Malformation: Management Evolution and Technical Innovation. Childs Nerv. Syst..

[10] Iskandar B.J., Hedlund G.L., Grabb P.A., Oakes W.J. (1998). The Resolution of Syringohydromyelia without Hindbrain Herniation after Posterior Fossa Decompression. J. Neurosurg..

[11] Morgenstern P.F., Tosi U., Uribe-Cardenas R., Greenfield J.P. (2020). Ventrolateral Tonsillar Position Defines Novel Chiari 0.5 Classification. World Neurosurg..

[12] Tubbs R.S., Iskandar B.J., Bartolucci A.A., Oakes W.J. (2004). A Critical Analysis of the Chiari 1.5 Malformation. J. Neurosurg..

[13] Fisahn C., Shoja M.M., Turgut M., Oskouian R.J., Oakes W.J., Tubbs R.S. (2016). The Chiari 3.5 Malformation: A Review of the Only Reported Case. Childs Nerv. Syst..

[14] Tubbs R.S., Muhleman M., Loukas M., Oakes W.J. (2012). A New Form of Herniation: The Chiari V Malformation. Childs Nerv. Syst..

[15] Gardner W.J., Goodall R.J. (1950). The Surgical Treatment of Arnold-Chiari Malformation in Adults; an Explanation of Its Mechanism and Importance of Encephalography in Diagnosis. J. Neurosurg..

[16] Oldfield E.H., Muraszko K., Shawker T.H., Patronas N.J. (1994). Pathophysiology of Syringomyelia Associated with Chiari I Malformation of the Cerebellar Tonsils. Implications for Diagnosis and Treatment. J. Neurosurg..

[17] Nishikawa M., Sakamoto H., Hakuba A., Nakanishi N., Inoue Y. (1997). Pathogenesis of Chiari Malformation: A Morphometric Study of the Posterior Cranial Fossa. J. Neurosurg..

[18] Goel A. (2015). Is Atlantoaxial Instability the Cause of Chiari Malformation? Outcome Analysis of 65 Patients Treated by Atlantoaxial Fixation. J. Neurosurg. Spine.

[19] Kim I.-K., Wang K.-C., Kim I.-O., Cho B.-K. (2010). Chiari 1.5 Malformation: An Advanced Form of Chiari I Malformation. J. Korean Neurosurg. Soc..

[20] Attenello F.J., McGirt M.J., Gathinji M., Datoo G., Atiba A., Weingart J., Carson B., Jallo G.I. (2008). Outcome of Chiari-Associated Syringomyelia after Hindbrain Decompression in Children: Analysis of 49 Consecutive Cases. Neurosurgery.

[21] Grenier-Chartrand F., Taverne M., James S., Guida L., Paternoster G., Loiselet K., Beccaria K., Dangouloff-Ros V., Levy R., de Saint Denis T. (2023). Mobility Assessment Using Multi-Positional MRI in Children with Cranio-Vertebral Junction Anomalies. J. Clin. Med..

[22] Ball M.J., Dayan A.D. (1972). Pathogenesis of Syringomyelia. Lancet.

[23] Tubbs R.S., Hill M., Loukas M., Shoja M.M., Oakes W.J. (2008). Volumetric Analysis of the Posterior Cranial Fossa in a Family with Four Generations of the Chiari Malformation Type I. J. Neurosurg. Pediatr..

[24] Greenberg J.K., Milner E., Yarbrough C.K., Lipsey K., Piccirillo J.F., Smyth M.D., Park T.S., Limbrick D.D. (2015). Outcome Methods Used in Clinical Studies of Chiari Malformation Type I: A Systematic Review. J. Neurosurg..

[25] Roller L.A., Bruce B.B., Saindane A.M. (2015). Demographic Confounders in Volumetric MRI Analysis: Is the Posterior Fossa Small in the Adult Chiari 1 Malformation?. AJR Am. J. Roentgenol..

[26] Sgouros S., Williams B. (1995). A Critical Appraisal of Drainage in Syringomyelia. J. Neurosurg..

[27] Almotairi F.S., Hellström P., Skoglund T., Nilsson Å.L., Tisell M. (2020). Chiari I Malformation-Neuropsychological Functions and Quality of Life. Acta Neurochir..

[28] Bianchi F., Benato A., Frassanito P., Tamburrini G., Massimi L. (2021). Functional and Morphological Changes in Hypoplasic Posterior Fossa. Childs Nerv. Syst..

[29] Visocchi M. (2020). Surgery and Neuropsychological Functions Changes in Chiari I Malformation: Two Faces of the Same Medal?. Acta Neurochir..

[30] Liao C., Visocchi M., Zhang W., Li S., Yang M., Zhong W., Liu P. (2019). The Relationship Between Basilar Invagination and Chiari Malformation Type I: A Narrative Review. Acta Neurochir. Suppl..

[31] Jian F.-Z., Chen Z., Wrede K.H., Samii M., Ling F. (2010). Direct Posterior Reduction and Fixation for the Treatment of Basilar Invagination With Atlantoaxial Dislocation. Neurosurgery.

[32] Menezes A.H. (2008). Craniovertebral Junction Database Analysis: Incidence, Classification, Presentation, and Treatment Algorithms. Childs Nerv. Syst..

[33] Lou Y., Yang J., Wang L., Chen X., Xin X., Liu Y. (2019). The Clinical Efficacy Study of Treatment to Chiari Malformation Type I with Syringomyelia under the Minimally Invasive Surgery of Resection of Submeningeal Cerebellar Tonsillar Herniation and Reconstruction of Cisterna Magna. Saudi J. Biol. Sci..

[34] Salunke P., Karthigeyan M., Malik P., Panchal C. (2020). Changing Perception but Unaltered Reality: How Effective Is C1-C2 Fixation for Chiari Malformations without Instability?. World Neurosurg..

[35] Visocchi M., Trevisi G., Iacopino D., Tamburrini G., Caldarelli M., Barbagallo G. (2014). Odontoid Process and Clival Regeneration with Chiari Malformation Worsening after Transoral Decompression: An Unexpected and Previously Unreported Cause of “Accordion Phenomenon”. Eur. Spine J..

[36] Besteiro González J.L., Torres Campa-Santamarina J.M. (2018). Anomalies in the Cognitive-Executive Functions in Patients with Chiari Malformation Type I. Psicothema.

[37] Das S., Stone L., Godzik J., Kelly M. (2023). Spine Deformity Associated with Chiari I Malformation and Syringomyelia. Neurosurg. Clin. N. Am..

[38] García M., Lázaro E., López-Paz J.F., Martínez O., Pérez M., Berrocoso S., Al-Rashaida M., Amayra I. (2018). Cognitive Functioning in Chiari Malformation Type I Without Posterior Fossa Surgery. Cerebellum.

[39] Lavorato L., Spallone A., Visocchi M. (2019). Surgical Treatment of Chiari Malformation in Adults: Comparison of Surgical Techniques Described in the Literature and Our Experience. Acta Neurochir. Suppl..

[40] Samantray S., Silva A.H.D., Valetopoulou A., Tahir Z. (2023). Foramen Magnum Decompression with Cervical Syringotomy for Chiari Malformation Type I with Syringomyelia—A Useful Adjunct in Selected Cases. Surg. Neurol. Int..

[41] Chang T., Zhang X., Maoliti W., Yuan Q., Yang X., Wang J. (2021). Outcomes of Dura Splitting Decompression Versus Posterior Fossa Decompression With Duraplasty in the Treatment of Chiari I Malformation: A Systematic Review and Meta-Analysis. World Neurosurg..

[42] Ciappetta P., Signorelli F., Visocchi M. (2019). The Role of Arachnoid Veils in Chiari Malformation Associated with Syringomyelia. Acta Neurochir. Suppl..

[43] Ellenbogen R.G., Armonda R.A., Shaw D.W., Winn H.R. (2000). Toward a Rational Treatment of Chiari I Malformation and Syringomyelia. Neurosurg. Focus.

[44] Villa A., Imperato A., Maugeri R., Visocchi M., Iacopino D.G., Francaviglia N. (2019). Surgical Treatment in Symptomatic Chiari Malformation Type I: A Series of 25 Adult Patients Treated with Cerebellar Tonsil Shrinkage. Acta Neurochir. Suppl..

[45] Bauer D.F., Pattisapu J.V., Ackerman L.L., Infinger L.K., Jackson E.M., Jernigan S., Maher C.O., Niazi T., Qaiser R., Quinsey C. (2024). In Reply: Congress of Neurological Surgeons Systematic Review and Evidence-Based Guidelines for Patients with Chiari Malformation: Surgical Interventions. Clin. Neurosurg..

[46] Di Lorenzo N., Palma L., Palatinsky E., Fortuna A. (1995). “Conservative” Cranio-Cervical Decompression in the Treatment of Syringomyelia-Chiari I Complex. A Prospective Study of 20 Adult Cases. Spine.

[47] Sahuquillo J., Rubio E., Poca M.A., Rovira A., Rodriguez-Baeza A., Cervera C. (1994). Posterior Fossa Reconstruction: A Surgical Technique for the Treatment of Chiari I Malformation and Chiari I/Syringomyelia Complex--Preliminary Results and Magnetic Resonance Imaging Quantitative Assessment of Hindbrain Migration. Neurosurgery.

[48] Spennato P., Vitulli F., Tafuto R., Imperato A., Mirone G., Cinalli G. (2023). Fourth Ventricle to Spinal Subarachnoid Space Stenting in Pediatric Patients with Refractory Syringomyelia: Case Series and Systematic Review. Neurosurg. Rev..

[49] Batzdorf U., Klekamp J., Johnson J.P. (1998). A Critical Appraisal of Syrinx Cavity Shunting Procedures. J. Neurosurg..

[50] Aydin L., Dereli D., Kartum T.A., Sirinoglu D., Sahin B., Eksi M.S., Musluman A.M., Yilmaz A. (2024). Management of Persistent Syringomyelia in Patients Operated for Chiari Malformation Type 1. World Neurosurg..

[51] Logue V., Edwards M.R. (1981). Syringomyelia and Its Surgical Treatment--an Analysis of 75 Patients. J. Neurol. Neurosurg. Psychiatry.

[52] Matsumoto T., Symon L. (1989). Surgical Management of Syringomyelia--Current Results. Surg. Neurol..

[53] Mazzola C.A., Fried A.H. (2003). Revision Surgery for Chiari Malformation Decompression. Neurosurg. Focus.

[54] Soleman J., Roth J., Constantini S. (2019). Syringo-Subarachnoid Shunt: How I Do It. Acta Neurochir..

[55] Massimi L., Pennisi G., Frassanito P., Tamburrini G., Di Rocco C., Caldarelli M. (2019). Chiari Type I and Hydrocephalus. Childs Nerv. Syst..

[56] Tubbs R.S., McGirt M.J., Oakes W.J. (2003). Surgical Experience in 130 Pediatric Patients with Chiari I Malformations. J. Neurosurg..

[57] Massimi L., Pravatà E., Tamburrini G., Gaudino S., Pettorini B., Novegno F., Colosimo C., Di Rocco C. (2011). Endoscopic Third Ventriculostomy for the Management of Chiari I and Related Hydrocephalus: Outcome and Pathogenetic Implications. Neurosurgery.

[58] McGirt M.J., Nimjee S.M., Floyd J., Bulsara K.R., George T.M. (2005). Correlation of Cerebrospinal Fluid Flow Dynamics and Headache in Chiari I Malformation. Neurosurgery.

[59] Tubbs R.S., Bui C.J., Rice W.C., Loukas M., Naftel R.P., Holcombe M.P., Oakes W.J. (2007). Critical Analysis of the Chiari Malformation Type I Found in Children with Lipomyelomeningocele. J. Neurosurg..

[60] Selden N.R., Nixon R.R., Skoog S.R., Lashley D.B. (2006). Minimal Tethered Cord Syndrome Associated with Thickening of the Terminal Filum. J. Neurosurg..

[61] Massimi L., Peraio S., Peppucci E., Tamburrini G., Di Rocco C. (2011). Section of the Filum Terminale: Is It Worthwhile in Chiari Type I Malformation?. Neurol. Sci..

[62] Tubbs R.S., Loukas M., Shoja M.M., Oakes W.J. (2007). Observations at the Craniocervical Junction with Simultaneous Caudal Traction of the Spinal Cord. Childs Nerv. Syst..

[63] Royo-Salvador M.B., Solé-Llenas J., Doménech J.M., González-Adrio R. (2005). Results of the Section of the Filum Terminale in 20 Patients with Syringomyelia, Scoliosis and Chiari Malformation. Acta Neurochir..

[64] Milano J.B., Barcelos A.C.E.S., Onishi F.J., Daniel J.W., Botelho R.V., Dantas F.R., Neto E.R., de Freitas Bertolini E., Mudo M.L., Brock R.S. (2020). The Effect of Filum Terminale Sectioning for Chiari 1 Malformation Treatment: Systematic Review. Neurol. Sci..

[65] Gandhi R.H., German J.W. (2013). Minimally Invasive Approach for the Treatment of Intradural Spinal Pathology. 2013, 35, E5. Neurosurg. Focus.

[66] Ratre S., Yadav N., Yadav Y.R., Parihar V.S., Bajaj J., Kher Y. (2018). Endoscopic Management of Arnold-Chiari Malformation Type I with or without Syringomyelia. J. Neurol. Surg. A Cent. Eur. Neurosurg..

[67] Costa M., Avila M.J., Vivanco-Suarez J., Karas P., Monteith S., Patel A. (2024). Minimally Invasive Technique for Chiari I Decompression Without Durotomy: Surgical Technique and Preliminary Case Series. World Neurosurg..

[68] Kotil K., Ozdogan S., Kayaci S., Duzkalir H.G. (2018). Long-Term Outcomes of a New Minimally Invasive Approach in Chiari Type 1 and 1.5 Malformations: Technical Note and Preliminary Results. World Neurosurg..

[69] Zagzoog N., Reddy K.K. (2019). Use of Minimally Invasive Tubular Retractors for Foramen Magnum Decompression of Chiari Malformation: A Technical Note and Case Series. World Neurosurg..

[70] Tyagi G., Uppar A.M., Pruthi N., Shukla D. (2023). Minimally Invasive Foramen Magnum Decompression Using Tubular Retractors (MIFT) for Chiari I Malformations. Acta Neurochir. (Wien).

[71] Di X. (2009). Endoscopic Suboccipital Decompression on Pediatric Chiari Type I. Minim. Invasive Neurosurg..

[72] Deng K., Li Y.-N., Li G.-L., Gao J., Yang Z., Di X., Wang R.-Z. (2010). Neural Endoscopic Assisted Micro-Invasive Management of Chiari I Malformation. Chin. Med. J..

[73] Pakzaban P. (2017). Technique for Mini-Open Decompression of Chiari Type I Malformation in Adults. Oper. Neurosurg..

[74] Khalessi A.A., Rahme R., Rennert R.C., Borgas P., Steinberg J.A., White T.G., Santiago-Dieppa D.R., Boockvar J.A., Hatefi D., Pannell J.S. (2019). First-in-Man Clinical Experience Using a High-Definition 3-Dimensional Exoscope System for Microneurosurgery. Oper. Neurosurg..

[75] Mattogno P.P., Della Pepa G.M., Menna G., Agostini L., Albanese A., Tamburrini G., Gaudino S., Olivi A., Doglietto F. (2024). Posterior Cranial Fossa Surgery with a 3 Dimensional Exoscope: A Single-Center Survey-Based Analysis and a Literature Review. World Neurosurg..

